# Prostatic artery embolization in men with severe hemophilia a: a case report of two patients

**DOI:** 10.1186/s42155-022-00299-x

**Published:** 2022-04-21

**Authors:** Petra Svarc, Peter Kampmann, Lars Lönn, Martin Andreas Røder

**Affiliations:** 1 Department of Radiology, Rigshospitalet, Blegdamsvej 9, 2100 Copenhagen, Denmark; 2 Department of Clinical Medicine, Faculty of Health and Medical Sciences, University of Copenhagen, Blegdamsvej 3B, 2100 Copenhagen, Denmark; 3 Department of Hematology, Rigshospitalet, Blegdamsvej 9, 2100 Copenhagen, Denmark; 4 Copenhagen Prostate Cancer Center, Department of Urology, Rigshospitalet, Blegdamsvej 9, 2100 Copenhagen, Denmark

**Keywords:** Hemophilia a, Prostatic artery embolization, Benign prostatic hyperplasia

## Abstract

**Background:**

This is the first case report describing the peri- and postoperative hemostasis plans in two men with severe hemophilia A (HA) who underwent prostatic artery embolization (PAE) for symptomatic benign prostatic hyperplasia (BPH).

**Case presentation:**

Two patients with severe HA and lower urinary tract symptoms (LUTS) not responding to medical therapy underwent PAE at our institution. In both patients, intermittent administration of decreasing doses of extended half-life recombinant factor VIII (EHL rFVIII) concentrate from 30 min before to 7 days after the PAE resulted in good hemostatic control. In addition to EHL rFVIII, tranexamic acid was administered in the same timeframe to augment the action of EHL rFVIII and to account for possible mucosal bleeding from the urinary tract. Both patients reported a minor localized hematoma at the femoral puncture site in the right groin, that resolved spontaneously. No other bleeding complications were observed.

**Conclusions:**

The procoagulant effects of the chosen dosing of EHL rFVIII showed sufficient to perform a technically successful embolization. At 6 months follow-up, both patients had significant reduction in self-reported urinary symptoms and were content with the outcome.

## Background

Hemophilia A (HA) is a X-linked recessive disorder resulting in a congenital deficiency of factor VIII (FVIII), with a worldwide incidence of around 1 in 5000 live male births (Iorio et al., 2019). As a result of advances in treatment over the past decades, life expectancy of patients with severe HA is approaching that of the general population (Hassan et al., 2021). Consequently, chronic conditions related to aging, such as benign prostatic hyperplasia (BPH), are increasingly present in the patient population. BPH is a frequent cause of lower urinary tract symptoms (LUTS), which include increased frequency of urination, nocturia, hesitancy, urgency, and weak urinary stream. One fourth of men older than 70 years have moderate to severe LUTS that impair their quality of life (QOL) (Thorpe & Neal, 2003). Medical therapy is the first line of treatment for men with symptomatic BPH, but in a subset of men with persisting symptoms surgical options need to be considered (Lerner et al., 2021a; Lerner et al., 2021b). Transurethral resection of prostate (TURP) is the golden standard surgical treatment of BPH, but can be associated with significant blood loss, a major challenge in the setting of severe hemophilia (Kirollos & Campbell, 1997).

Prostatic artery embolization (PAE) is emerging as a minimally invasive alternative to TURP (Xiang et al., 2021). Similar to other endovascular procedures requiring arterial vascular access, an arterial puncture of the common femoral artery (CFA) is performed as the first step of the procedure. In addition to avoiding hemorrhagic adverse events, the hemostasis needs to be sufficient to form a clot in the target arteries for the embolization to be successful. Only sporadic case reports regarding the safety of endovascular procedures and optimal perioperative factor substitution in men with severe HA exist in the literature (Beirne et al., 2007; Marrocco-Trischitta et al., 2009; Garge et al., 2016). To our knowledge, no studies of patients with severe HA undergoing PAE have been published. We present two patients with severe HA who successfully underwent PAE without any bleeding episodes in the perioperative or early postoperative period.

## Case presentation

The first patient is a 60-year-old man with severe HA who was referred for PAE due to severe LUTS impacting his QOL. The Danish Prostatic Symptom Score (DAN-PSS) was 24 (Schou et al., 1993). Transrectal ultrasound (TRUS) showed a prostate volume of 131 cm^3^. Previous urological treatment included an alfa-blocker in combination with finasteride. Uroflowmetry demonstrated urinary obstruction with a maximum flow of 11.6 ml/s. The medical history included essential hypertension, osteoporosis, and transfusion-acquired chronic HIV infection. His standard factor prophylaxis constitutes of self-administering 3000 IU of extended half-life recombinant factor VIII (EHL rFVIII) concentrate (efmoroctocog alfa) every third day. He has no factor inhibitor present.

The second patient, a 53-year-old man with severe HA and Factor V Leyden mutation, was likewise referred to a consultant urologist due to LUTS not responding to standard medical therapy. Prostate volume on TRUS was 44 cm^3^, and he had a DAN-PSS of 30 points, defined as severe LUTS. Uroflowmetry showed maximum flow of 17.4 ml/s, with an obstructive curve. Other relevant medical history included essential hypertension and osteopenia. His standard prophylaxis consists of self-administering 4000 IU of EHL rFVIII concentrate (ruriotocog alfa pegol) every fifth day, and no inhibitor is present.

Hemostasis plans for peri- and postoperative management were made by a consultant hematologist from the patients’ Comprehensive Hemophilia Care Centre and included intermittent administration of EHL rFVIII and tranexamic acid (TA) for 7 days following PAE, as seen in Table 1.

**Table 1:** Perioperative hemostasis regimens for both patients

Timing	EHL rFVIII (IU)^a^		Tranexamic acid (mg)^b^	
	Patient 1	Patient 2	Patient 1	Patient 2
Day 0				
30 min before PAE	4000	4000	1000	1000
6 h after PAE	1000	/	/	/
8 h after PAE	/	2000	/	/
Day 1–3	2000	2000	3 × 1000	3 × 1000
Day 4–7	1000	1000	3 × 1000	3 × 1000
Day 8	Standard prophylaxis is resumed			

^a^Both patients received their standard FVIII preparations. ^b^ Tranexamic acid was given intravenously on Day 0, and perorally on the following days

*PAE* Prostatic artery embolisation*, EHL rFVIII* Extended Half Life Recombinant Factor VIII

Initial (preoperative) dose of both EHL rFVIII and TA was timed at 30 min before procedure start to provide maximal coverage at the time of the greatest bleeding risk and the highest need for procoagulant therapeutic effect during the embolization process. Blood sample was taken immediately after factor administration to assess if optimal FVIII activity was achieved.

**Fig. 1:**
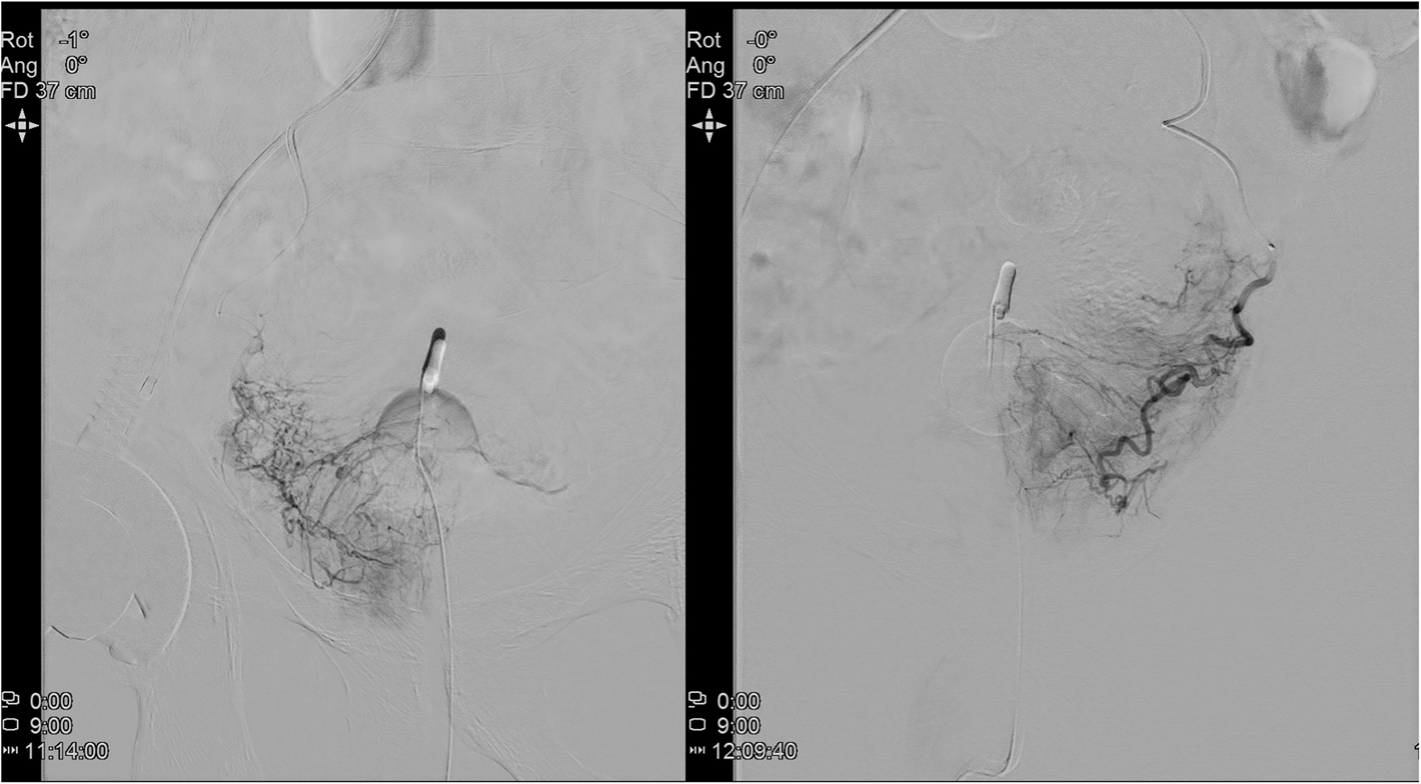
Selective AP angiograms in Patient 1 with the microcatheter in the right (left panel) and left (right panel) prostatic artery and contrast blush of the corresponding prostate side

**Fig. 2:**
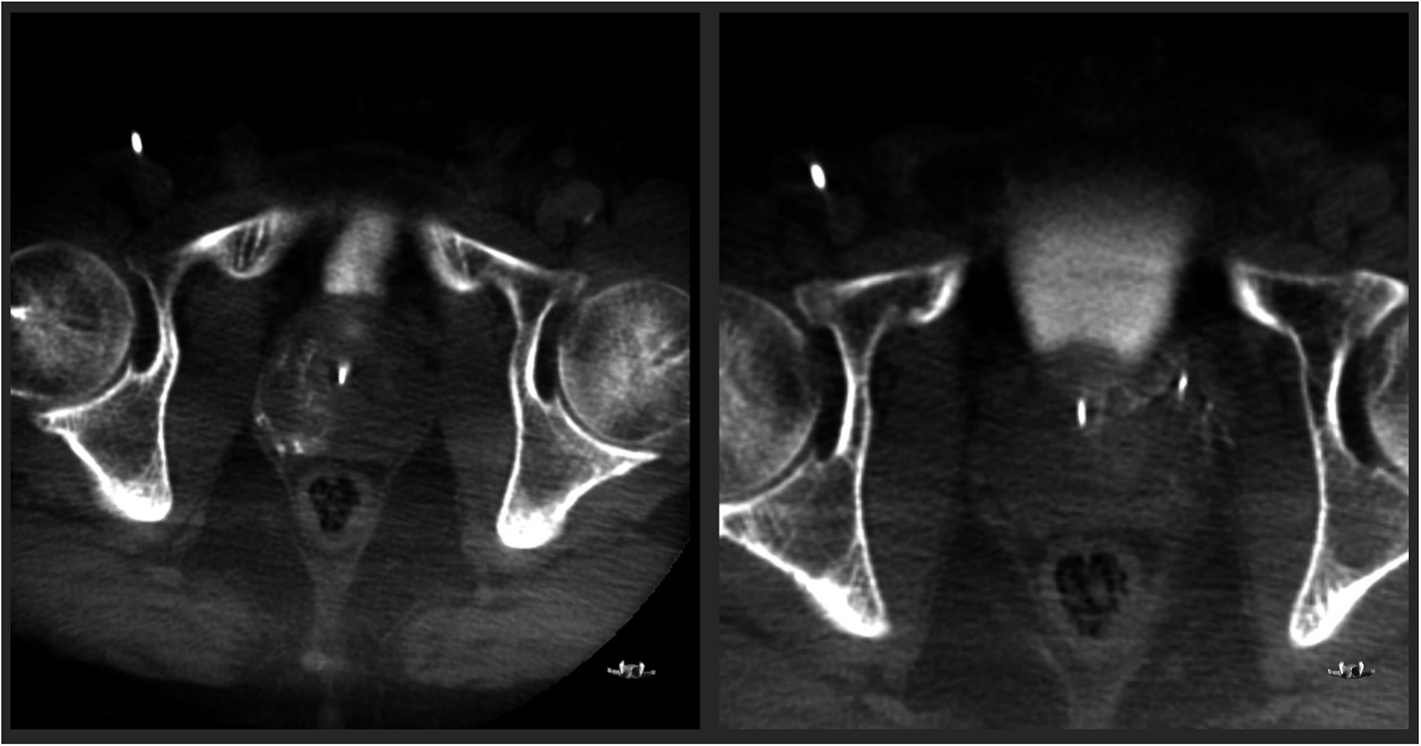
Cone beam CT (CBCT) images from Patient 2 used for confirmation of microcatheter position show contrast enhancement in right (left panel) and left (right panel) prostate sides. No enhancement is seen in the neighboring structures

In an angio-suite, arterial vascular access for PAE was achieved via puncture of the right CFA with a 19G needle accommodating a 0.035-in. guidewire, and subsequent introduction of a 6 Fr sheath to maintain access, as per operator preference. Under direct fluoroscopic guidance the desired catheter position was reached and evaluated for prostatic enhancement and collaterals (Fig. 1). Cone beam CT (CBCT) was used for further confirmation of microcatheter position (Fig. 2). PAE was performed using the standard proximal embolization first, followed by distal (PErFecTED) technique (Carnevale et al., 2014). Microspheres (Embosphere 300–500 μm, Merit Medical) were injected to achieve arterial occlusion of both prostate sides. Puncture site hemostasis was successfully achieved in both patients by using the Angio-Seal (Terumo) vascular closure device. The patients were advised to take bed rest for 2 h following PAE. An additional EHL rFVIII dose was given 6 (Patient 1) or 8 h (Patient 2) following arterial closure. Both patients were discharged from the hospital the following morning. For the following 7 days gradually decreasing doses of EHL rFVIII were administrated once daily, while 1000 mg of TA was administered three times daily. Finally, standard factor prophylaxis was resumed in both patients on the eighth day. Screening for FVIII inhibitor was negative in both patients.

Patient 1 reported symptoms of the postembolisation syndrome (fever, pelvic pain, and dysuria) lasting for the first 10 days following PAE. Both patients reported a small, localized hematoma around the puncture site in the right groin, that resolved spontaneously. No other bleeding complications were observed. Both patients reported an improvement in their LUTS symptoms. Patient 1 had a DAN-PSS of 3 points after 6 months and no further medical treatment for LUTS. Patient 2 reported zero symptoms after 6 months follow-up. Medical records noted both patients were satisfied with the result.

## Conclusions

We successfully embolized the prostate in two men with HA for severe LUTS and BPH with no hemorrhagic adverse events and good clinical effect. To our knowledge, this is the first report of a novel treatment for BPH in men who are often rejected for surgery due to the high risk of surgical complications.

BPH can manifest with a variety of symptoms. In addition to LUTS, microscopic or gross hematuria is present in approximately 2.5% of patients without bleeding disorders (Tapping et al., 2018). There are no studies investigating the incidence of spontaneous hematuria in patients with hemophilia, but a link between anticoagulation and increased hematuria rates has been well-described in the literature (Wallis et al., 2017). BPH-related hematuria can result in blood loss requiring transfusion, especially as the prostate volume increases and developing optimal management strategies for patients with HA is of clinical importance.

TURP is still regarded as the golden standard in the surgical treatment of BPH, but it is associated with higher complication rates compared to newer minimally invasive treatments (Rassweiler et al., 2006). In a recent study, TURP was associated with increased risk of bleeding in patients with HA, particularly after the patients have returned home (Mesnard et al., 2021). PAE is rapidly being established as a robust and safe alternative to surgery, especially in patients with larger prostates. In contrast to TURP and other minimally invasive alternatives, PAE does not involve direct manipulation of prostate tissue, which results in fewer hemorrhagic adverse events (Moreira et al., 2017). This could make PAE the procedure of choice in patients with bleeding disorders.

Both patients were on a routine prophylactic regimen of 4000 IU EHL rFVIII, administered every 72 h for Patient 1 and every 96 h for Patient 2. Pharmacokinetic profiles of EHL rFVIII were known for both patients. The hemostatic regimens we designed aimed at maintaining FVIII level of 0.8–1.0 kIU/L for the first 24 h, and gradually lowering the target levels to 0.4–0.6 kIU/L for the following 6 days. The rationale for this decision was to secure hemostatic capability comparable to that of the general male population in which there is previous PAE experience.

Literature concerning the safety of endovascular procedures and optimal factor replacement strategies in patients with severe HA is sparse. Guidelines for factor replacement in surgery in severe HA refer to general surgery, with no specific mention of endovascular procedures (Srivastava et al., 2020). Few factor replacement protocols in endovascular procedures exist, but good outcomes with intermittent factor replacement strategies have been published in case reports of patients undergoing transjugular intrahepatic portosystemic shunt (TIPS) placement and endovascular abdominal aortic repair (EVAR), though no prospective trials investigating the optimal approach exist (Beirne et al., 2007; Marrocco-Trischitta et al., 2009). The general approach used in the published literature is to maintain a factor level of between 50 and 100% throughout the procedure, in addition to any antithrombotic agents as per existing guidelines for the normal population. In our experience, intermittent factor replacement provides satisfactory factor levels while helping to minimize the overall cost. Following PAE, transient hematuria and hematospermia can occur as minor complications in about 10% of patients (Moreira et al., 2017; Zhang et al., 2018). To account for that, TA was added to the management plans as an antifibrinolytic agent with good effect.

To conclude, further studies are needed to investigate the optimal regimens in patients with severe HA undergoing PAE, but intermittent factor replacement seems to provide good hemostasis in the perioperative period.

## Data Availability

Data sharing is not applicable to this article as no datasets were generated or analysed during the current study.

## Abbreviations

BPH: Benign prostatic hyperplasia
CBCT: Cone beam CT
CFA: Common femoral artery
DAN-PSS: Danish Prostatic Symptom Score
EHL FVIII: Extended half-life factor VIII
EVAR: Endovascular aortic repair
FVIII: Factor VIII
HA: Hemophilia A
LUTS: Lower urinary tract symptoms
PAE: Prostatic artery embolization
PErFecTED: Proximal embolization first then embolize distal
QOL: Quality of life
TIPS: Transjugular intrahepatic portosystemic shunt
TA: Tranexamic acid
TRUS: Transrectal ultrasound
TURP: Transurethral resection of the prostate
